# “Hunger Would Kill us Instead of the Pandemic:” Elders’ Responses to COVID-19 in Ethiopia

**DOI:** 10.1093/geroni/igab046.2714

**Published:** 2021-12-17

**Authors:** Messay Kotecho, Margaret Adamek, Anduamlak Takele

**Affiliations:** 1 Addis Ababa University, Addis Ababa, Adis Abeba, Ethiopia; 2 Indiana University, INDIANAPOLIS, Indiana, United States; 3 Debre Markos University, Debre Markos, Dire Dawa, Ethiopia

## Abstract

The COVID-19 pandemic has posed unpredictable challenges globally. Urban elders in Global South nations are among the major population groups vulnerable to COVID-19. A qualitative case study design was used to uncover the challenges and sources of support for poor urban elders during COVID-19 lockdown in Ethiopia. Data were collected from 27 elders age 60 and above in Debre Markos Town via in-depth interviews and document review. Narrative data were analyzed using thematic data analysis. Four prominent themes were identified: 1. Food insecurity (“Hunger would kill us instead of COVID-19”), 2. Hopelessness (“Feeling hopeless and begging to die”), 3. Social isolation (“We prefer social support rather than food donations”) and 4. Gratitude (“Feeling thankful”). The physical distancing program introduced to contain the pandemic isolated many elders and diminished their capacity to access support from others needed to perform their daily activities. An institutional welfare system is needed to ensure older adults in the Global South can live a joyful and dignified life, even through a global pandemic. Moreover, a special emergency fund to meet older adults’ basic needs during a pandemic like COVID-19 should be introduced to minimize the effect of crises on vulnerable groups like destitute older adults in Ethiopia.

